# Bazedoxifene, a selective estrogen receptor modulator, reduces cerebral aneurysm rupture in Ovariectomized rats

**DOI:** 10.1186/s12974-017-0966-7

**Published:** 2017-10-02

**Authors:** Hidetsugu Maekawa, Yoshiteru Tada, Kenji Yagi, Takeshi Miyamoto, Keiko T. Kitazato, Masaaki Korai, Junichiro Satomi, Tomoki Hashimoto, Shinji Nagahiro

**Affiliations:** 1 Department of Neurosurgery, Institute of Biomedical Sciences, Tokushima University Graduate School, 3-18-15 Kuramoto-cho, Tokushima, 770-8503 Japan; 2 Department of Anesthesia and Perioperative Care, University of California, San Francisco, 1001 Potrero Ave, SFGH 1, San Francisco, CA 94110 USA

**Keywords:** Estrogen, Estrogen receptor, Intracranial aneurysm, Selective estrogen receptor modulator, Subarachnoid hemorrhage

## Abstract

**Background:**

Estrogen deficiency is thought to be responsible for the higher frequency of aneurysmal subarachnoid hemorrhage in post- than premenopausal women. Estrogen replacement therapy appears to reduce this risk but is associated with significant side effects. We tested our hypothesis that bazedoxifene, a clinically used selective estrogen receptor (ER) modulator with fewer estrogenic side effects, reduces cerebral aneurysm rupture in a new model of ovariectomized rats.

**Methods:**

Ten-week-old female Sprague-Dawley rats were subjected to ovariectomy, hemodynamic changes, and hypertension to induce aneurysms (ovariectomized aneurysm rats) and treated with vehicle or with 0.3 or 1.0 mg/kg/day bazedoxifene. They were compared with sham-ovariectomized rats subjected to hypertension and hemodynamic changes (HT rats). The vasoprotective effects of bazedoxifene and the mechanisms underlying its efficacy were analyzed.

**Results:**

During 12 weeks of observation, the incidence of aneurysm rupture was 52% in ovariectomized rats. With no effect on the blood pressure, treatment with 0.3 or 1.0 mg/kg/day bazedoxifene lowered this rate to 11 and 17%, almost the same as in HT rats (17%). In ovariectomized rats, the mRNA level of ERα, ERβ, and the tissue inhibitor of metalloproteinase-2 was downregulated in the cerebral artery prone to rupture at 5 weeks after aneurysm induction; the mRNA level of interleukin-1β and the matrix metalloproteinase-9 was upregulated. In HT rats, bazedoxifene restored the mRNA level of ERα and ERβ and decreased the level of interleukin-1β and matrix metalloproteinase-9. These findings suggest that bazedoxifene was protective against aneurysmal rupture by alleviating the vascular inflammation and degradation exacerbated by the decrease in ERα and ERβ.

**Conclusions:**

Our observation that bazedoxifene decreased the incidence of aneurysmal rupture in ovariectomized rats warrants further studies to validate this response in humans.

**Electronic supplementary material:**

The online version of this article (10.1186/s12974-017-0966-7) contains supplementary material, which is available to authorized users.

## Background

The rupture of cerebral aneurysms elicits devastating subarachnoid hemorrhage (SAH). Surgical and endovascular treatments can be preventive but are associated with inherent risks and complications [1]. Therefore, medical therapy with lower risks for complications is needed to prevent SAH.

The incidence of cerebral aneurysms and SAH is higher in post- than premenopausal women [2, 3]. Post-menopausal estrogen deficiency is thought to play a key role in the pathophysiology of cerebral aneurysms. Our group established a cerebral aneurysm model in ovariectomized rats subjected to hemodynamic stress and hypertension [4]. The model mimics human postmenopausal hormonal conditions. Estrogen-deficient model rats had a higher incidence of cerebral aneurysms than male rats, and estrogen deficiency promoted endothelial damage and vascular inflammation [5–8]. Estrogen (17β-estradiol) and an estrogen receptor β (ERβ)-, but not an ERα agonist, reduced the incidence of cerebral aneurysm rupture in ovariectomized mice [9]. These findings suggest that ER-related drugs may help to prevent SAH. In humans, postmenopausal estrogen replacement therapy reduced the risk of SAH [10–12] but increased the risk for breast and endometrial cancer, stroke, and venous thromboembolism [9, 13–15].

Bazedoxifene (BZA), a selective estrogen receptor modulator (SERM), is used to prevent postmenopausal osteoporosis. It has fewer estrogenic side effects than conjugated estrogen. SERM is a ligand of ERα and ERβ; its characteristics are different from pure ER agonists and ER-antagonists because, depending on the target tissue, it exerts agonistic or antagonistic effects [16]. Its tissue-specific effects are thought to be attributable to its distinct affinity for each ER, a unique conformational change in ERs upon binding to SERM, and the distinct distribution of ERα and ERβ in the tissue [16, 17]. Therefore, we used our rat model to investigate whether BZA may represent a potential and safer prophylactic agent against cerebral aneurysm rupture in postmenopausal women.

We established a novel rat model of aneurysm rupture by modifying the carotid ligation procedure used in our original model in which ovariectomized rats were subjected to hemodynamic changes and hypertension without pharmacologically elicited degeneration of the arterial wall [18]. The pathological features of the experimental aneurysms in our ovariectomized hypertensive rats were those of human aneurysms. In the posterior cerebral artery (PCA) where ruptured aneurysms formed most frequently, the mRNA level of interleukin (IL)-1β was higher and the imbalance of matrix metalloproteinase (MMP)-9 and the level of the tissue inhibitor of metalloproteinase-2 (TIMP-2) were greater than at the bifurcation of the anterior cerebral artery-olfactory artery (ACA-OA) where no ruptured aneurysms were observed. We suggested that these molecules associated with inflammation and vascular degradation are at least partly responsible for aneurysmal rupture [18]. Here, we used our modified rat aneurysm model to test the hypothesis that BZA exerts protective effects against vascular inflammation and aneurysm rupture.

We now demonstrate that BZA reduced the incidence of aneurysmal rupture without affecting the blood pressure in ovariectomized aneurysm-model rats. Its restoration of the decreased expression of both ERs in the aneurysm-prone cerebral artery was associated with the reduction of pro-inflammatory cytokines and vascular degradation molecules. We also show that BZA attenuated the body weight gain seen under conditions of estrogen deficiency and hypertension. Our findings suggest BZA as a potential therapeutic agent for the prevention of SAH in postmenopausal women.

## Methods

### Animals

Sexually mature (10-week-old) [19] female Sprague-Dawley rats (220–250 g, *n* = 137; Charles River Laboratories Japan Inc., Yokohama, Japan) were housed under specific-pathogen-free conditions and controlled temperatures (23 ± 2 °C), humidity (55 ± 10%), and light (12-h light/dark cycle, 8 am–8 pm). Food and water were *ad libitum*.

### Cerebral aneurysm induction

Cerebral aneurysms were induced by hypertension (renal artery ligation and a high-salt diet), increased hemodynamic changes (carotid artery ligation), and estrogen deficiency (bilateral ovariectomy) as described elsewhere [18]. We ligated the right external carotid and the pterygopalatine artery and the left common carotid artery. The rats were bilaterally ovariectomized immediately after carotid ligation and then fed a high-salt diet (8% sodium chloride). Two weeks later, the bilateral posterior renal arteries were ligated.

### Experimental study 1

To assess whether ovariectomy increases and BZA treatment reduces the incidence of cerebral aneurysm rupture in hypertensive ovariectomized rats, 84 rats were randomly assigned to three groups (each group *n* = 28). One group was treated with vehicle (HT+OVX); the other two groups received 0.3 or 1.0 mg/kg/day BZA (BZA0.3, BZA1, respectively). Another set of 28 rats was sham-ovariectomized, subjected to hypertension, exposed to hemodynamic changes, and treated with vehicle (HT rats). BZA was kindly provided by Pfizer Inc. (New York, NY, USA). The BZA dose, determined based on an earlier study [20], was suspended in 5% Arabic gum solution (BZA0.3 rats: 0.06 mg/ml, BZA1 rats: 0.2 mg/ml) and perorally administered once a day (5 ml/kg for all solutions) for 12 weeks by blinded investigators. The 5% Arabic gum solution was also the vehicle. In our previous study, cerebral aneurysms started to rupture approximately 30 days after their induction [18]. The administration of BZA and of vehicle was started 2 weeks after renal artery ligation so that BZA would affect the rupture rather than the formation of aneurysms.

Aneurysm rupture was suspected when the rats exhibited neurologically abnormal behavior [21], developed a significant weight loss (> 30 g/day, approximately 10% of their body weight), or died. Rats with these findings were sacrificed immediately, and their brains were inspected to confirm SAH due to rupture of a cerebral aneurysm. Blinded investigators recorded the body weight daily to detect a sudden weight loss possibly indicative of aneurysmal rupture, assessed the clinical signs, and evaluated the brains. A representative ruptured aneurysm at the left PCA is shown in Additional file 1: Figure S1. All surviving rats were euthanized 12 weeks after aneurysm induction, and their brains were inspected for the presence of ruptured aneurysms. Data collection and analysis were performed in blinded fashion.

Of the 112 rats, we excluded 14 (5 HT+OVX, 1 BZA0.3, 4 BZA1, and 4 HT rats) because there was no evidence of rupture, although aneurysmal rupture was suspected.

#### Blood pressure measurements

To measure the blood pressure, the rats were placed on a 37 °C hot plate (Nissin, Tokyo, Japan) and covered with a blanket. After they adapted to the environment, their systolic blood pressure was measured twice, and the mean value was recorded. Blood pressure measurements were obtained immediately before and 2 and 12 weeks after renal artery ligation by the tail-cuff, auto-pickup method (Softron, Tokyo, Japan).

### Experimental study 2

To analyze the molecular mechanisms underlying the effects of ovariectomy and BZA on rat cerebral aneurysms, we performed quantitative real-time PCR (qRT-PCR) to assess the gene expression level and immunohistochemical staining to determine the protein expression level in the cerebral artery.

#### qRT-PCR assay

Another set of 21 rats was randomly divided into three equal groups of HT+OVX, BZA1, and HT rats. Bazedoxifene or vehicle was administered as in the “Experimental study 1” section. We did not prepare a BZA0.3 group because experimental study 1 had shown that the incidence of cerebral aneurysm rupture was not significantly different between BZA0.3 and BZA1 rats. To avoid excluding rats that would develop aneurysmal rupture, we euthanized these rats at 5 weeks after renal ligation before their experimentally induced aneurysms had begun to rupture in experimental study 1. We collected tissue samples from these 21 rats from the left proximal PCA (between the basilar bifurcation and the posterior communicating artery) where ruptured cerebral aneurysms had formed most frequently in HT+OVX rats in experimental study 1.

After transcardial perfusion with normal saline, we removed the left PCA. Total RNA was extracted with the MagNA Pure RNA isolation kit (Roche, Tokyo, Japan) and placed in a MagNA lyser (Roche). We used Transcriptor Universal cDNA Master (Roche) for the reverse transcription of total RNA to cDNA and a LightCycler 2.0 (Roche Diagnostics, Tokyo, Japan) for qRT-PCR. LightCycler FastStart DNA Master and SYBR Green I (Roche) were used for glyceraldehyde 3-phosphate dehydrogenase (GAPDH), ERα, ERβ, IL-1β, IL-6, tumor necrosis factor-α (TNF-α), MMP-9, and TIMP-2. The primers were as follows: for GAPDH, forward (F), 5′-TAC ACT GAG GAC CAG GTT G-3′, reverse (R), 5′-CCC TGT TGC TGT AGC CAT A-3′; for ERα, F, 5′-TGC ACC ATC GAT AAG AAC C-3′, R, 5′-GTC TCC TGA AGT GCC CAT T-3′; for ERβ, F, 5′-CTG CAT GGC TGA GCG ACA A-3′, R, 5′-AGA GAC TCA TGG GAC TCA GAT-3′; for IL-1β, F, 5′-TGC AGG CTT CGA GAT GAA C-3′, R, 5′-AGC TCA TGG AGA ATA CCA CTT G-3′; for IL-6, F, 5′-TCT CAG GGA GAT CTT GGA AAT G-3′, R, 5′-TAG AAA CGG AAC TCC AGA C-3′; for TNF-α, F, 5′-CCC AAC AAG GAG AAG T-3′, R, 5′-CGC TTG GTG GTT TGC TAC-3′; for MMP-9, F, 5′-CCT GGA ACT CAC ACA ACG-3′, R, 5′-GAG GTC ATA GGT CAC GTA GG -3′; and for TIMP-2, F, 5′-CCC TCT GTG ACT TTA TTG TGC-3′, R, 5′-TGA TGC TCT CTG TGA CC-3′. The PCR conditions were 95 °C for 10 min followed by 40 cycles at 95 °C for 10 s, 60 °C for 10 s, and 72 °C for 8 s. We subjected samples from each group to two independent qRT-PCR assays to determine the gene expression level. The results were quantified after normalization to the expression of GAPDH mRNA. To assess the effect of BZA on the uterus, we collected the uterus and measured its weight.

#### Immunohistochemical staining

The left proximal PCA from two HT+OVX and two BZA1 rats was collected 5 weeks after renal ligation to confirm ER protein expression in the arterial wall. After perfusion with 4% paraformaldehyde, the left PCA was harvested, immersed in 4% paraformaldehyde (24 h at 4 °C), and successively dehydrated in 10, 20, and 30% sucrose. The arteries were rinsed with phosphate-buffered saline, embedded in OCT compound (Tissue-Tek Inc.), and cut into 5-μm thick serial sections with a cryotome (CM 1850; Leica). After 30-min serum-free protein blockade (Dako, Carpinteria, CA, USA), primary antibodies against ERα and ERβ (Santa Cruz Biotechnology, CA, USA) diluted 1:100 with Canget signal immunostain (Toyobo, Osaka, Japan) were added for overnight incubation at 4 °C. Sections not treated with the primary antibodies were the negative control. All sections were then incubated for 1 h at room temperature with fluorescein-conjugated secondary antibodies Alexa Fluor 594 (Molecular Probes, CA, USA) in Canget signal immunostain (1:800 dilution, Toyobo), mounted with Vectashield (Vector Laboratories, CA, USA), and examined under a fluorescence microscope (KEYENCE, BZ-X710, Osaka, Japan).

### Statistical analysis

For sample-size calculation, we implemented *α* = 0.05 and 1-β = 0.8 with an effect size of 0.4 (literature-based) [9] using G*Power 3.1 (University of Düsseldorf, Düsseldorf, Germany), indicating that 25 samples per group were required. Expecting a loss of 10–15% of the rats, we used 28 animals per group in experimental study 1. Statistical analyses were performed with SPSS 22 (IBM Armonk, NY). Fisher’s exact test was used to analyze the incidence of cerebral aneurysm rupture. Sequentially, obtained data (mean ± SD) were analyzed with analysis of variance (ANOVA) followed by Scheffe’s test for multiple group comparisons of the mRNA level. Differences were considered statistically significant at *p* < 0.05.

## Results

### BZA prevents aneurysmal rupture in rats

To determine whether estrogen deficiency increases while BZA decreases the incidence of cerebral aneurysm rupture in estrogen-deficient rats, we compared the incidence of rupture during 12 weeks of observation in hypertensive ovariectomized aneurysm rats treated with vehicle (HT+OVX), 0.3 mg/kg BZA (BZA0.3), or 1 mg/kg BZA (BZA1) and in hypertensive sham-ovariectomized rats (HT rats) (for their preparation see the “Experimental study 1” section). The treatments were started 2 weeks after bilateral renal ligation and continued throughout the 12-week observation period.

**Fig. 1:**
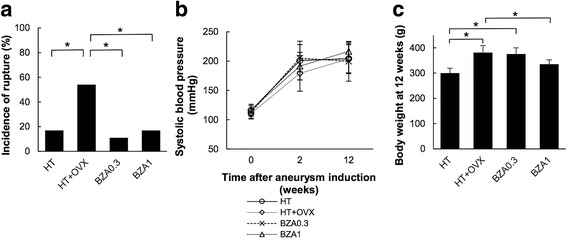
**a** Incidence of aneurysm rupture within 12 weeks after aneurysm induction (*p < 0.05, Fisher’s exact test). **b** Sequential changes in the systolic blood pressure (*p < 0.05 vs sham, Scheffe’s test). **c** The body weight at 12 weeks after aneurysm induction (*p < 0.05, Scheffe’s test). Data are the mean ± SD. *n* = 23 – 27 per group

As shown in Fig. 1a, HT+OVX rats manifested a significantly higher incidence of cerebral aneurysm rupture than HT rats (52 vs 17%, *p* < 0.05). BZA treatment decreased the incidence of rupture (BZA0.3, 11%; BZA1, 17%; each *p* < 0.05 vs HT+OVX rats). Notably, the rate of aneurysmal rupture in BZA0.3 and BZA1 rats was similar to that in HT rats, suggesting that the effect of BZA was not dose-dependent. BZA did not affect the systolic blood pressure, which was elevated after renal ligation (Fig. 1b). These results suggest that estrogen deficiency promoted aneurysm rupture in the presence of hypertension, while BZA inhibited its effect in a blood pressure-independent manner. Most ruptured aneurysms had arisen in the PCA (64%); 14% each were found in the internal carotid or the anterior cerebral artery (14%), and 9% in the middle cerebral artery.

Although there was no significant difference in the body weight at the time of randomization [HT, 251.8 ± 16.2 g; HT+OVX, 260.9 ± 22.7 g; BZA0.3, 261.0 ± 16.4 g; BZA1, 263.7 ± 12.7 g (mean ± SD)], 12 weeks after aneurysm induction, the body weight of HT+OVX rats was significantly greater than that of HT rats (381.4 ± 27.6 vs 300.2 ± 18.8 g, *p* < 0.05). The body weight of BZA1 was significantly lower than that of HT+OVX rats (263.7 ± 12.7 g vs 335.3 ± 17.3 g); treatment with 0.3 mg/kg BZA did not affect the body weight (375.8 ± 24.4 g) (Fig. 1c). In addition, 5 weeks after aneurysm induction, the wet uterine weight of HT+OVX rats was significantly lower than that of HT rats; it was similar in BZA1 and HT+OVX rats (HT, 440.5 ± 48.1 mg; HT + OVX, 145.5 ± 21.1 mg; BZA0.3, 261.0 ± 16.4 mg; BZA1, 134.7 ± 13.1 mg; mean ± SD; see Additional file 2: Figure S2]) The incidence of aneurysmal rupture and the body weight in BZA1 and HT rats were similar, suggesting that 1 mg/kg BZA revoked the effect of estrogen deficiency on the vasculature and the body weight without increasing the uterine weight.

### BZA upregulates ERα and ERβ expression in the cerebral artery

As BZA is a SERM acting on ERs, we next investigated how treatment with BZA affected the ER expression in the cerebral artery of estrogen-deficient rats. Five weeks after bilateral renal ligation, we assessed the mRNA level and the protein expression of ERs in the left PCA where ruptured aneurysms had formed most frequently in *experimental study 1*.

**Fig. 2:**
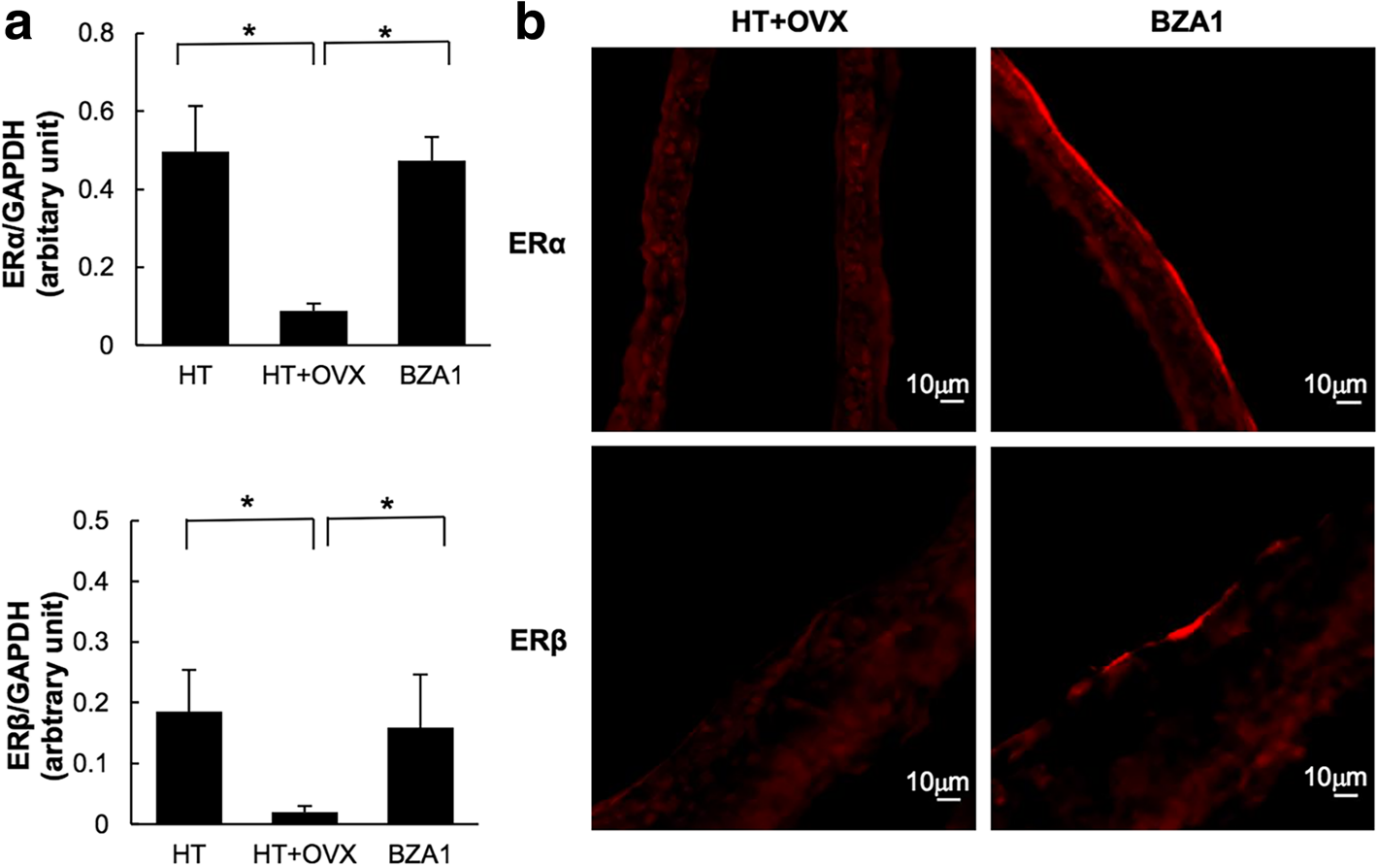
**a** mRNA level of estrogen receptor (ER)α and ERβ at the left posterior cerebral artery. The level was determined by quantitative real time-PCR and normalized by the glyceraldehyde 3-phophate dehydrogenase (GAPDH) mRNA level. *n* = 7 per group, *p < 0.05 (Scheffe’s test). Data are the mean ± SD. **b** Immunohistochemical staining for ERα and ERβ in the left posterior cerebral artery

HT+OVX rats exhibited significantly lower mRNA levels of ERα and ERβ than did HT rats with intact ovaries. In BZA1 rats, the mRNA level of both ERα and ERβ was significantly elevated and was similar to the level in HT rats (Fig. 2a). In agreement with the mRNA level of ERs, ERα, and ERβ, protein expression was low in the cerebral arteries of HT+OVX rats. In BZA1 rats, the expression of both ERα and ERβ was greater than in HT+OVX rats (Fig. 2b). These findings indicate that BZA restored the gene and protein expression of both ERα and ERβ that was decreased by estrogen deficiency.

### BZA downregulates IL-1β and decreases the MMP-9/TIMP-2 ratio in the cerebral artery

Elsewhere, we demonstrated that aneurysmal rupture in ovariectomized hypertensive rats was associated with the increase in the mRNA level of IL-1β and the MMP-9/TIMP-2 ratio in the aneurysmal wall [18, 22]. Here, we examined the mRNA level of IL-1β, IL-6, TNF-α, MMP-9, and TIMP-2 to explore the mechanisms underlying the protective effects of BZA against aneurysmal rupture.

**Fig. 3:**
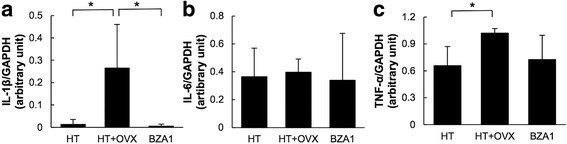
mRNA level of (**a**) interleukin (IL)-1β, (**b**) IL-6, (**c**) tumor necrosis factor (TNF)-α in the left posterior cerebral artery. The levels were determined by quantitative real time-PCR and normalized by the glyceraldehyde 3-phophate dehydrogenase (GAPDH) mRNA level. *p < 0.05 (Scheffe’s test). Data are the mean ± SD. *n* = 7 per group

The IL-1β mRNA level was significantly higher in HT+OVX than HT rats. In BZA1 rats, the level of IL-1β was significantly decreased and similar to the level in HT rats (Fig. 3a). The mRNA level of IL-6 was similar among HT, HT+OVX, and BZA1 rats (Fig. 3b). On the other hand, the mRNA level of TNF-α was significantly increased in HT+OVX rats, and while it was lower in BZA1 rats, the difference did not reach statistical significance (Fig. 3c). These findings suggest that the gene expression of IL-1β and TNF-α, but not of IL-6, is regulated at least partly by ERs.

**Fig. 4:**
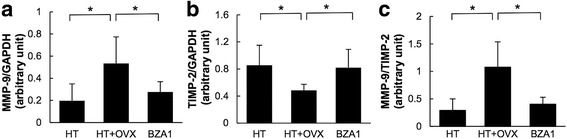
mRNA level of (**a**) matrix metalloproteinase (MMP)-9 and (**b**) tissue inhibitor of metalloproteinase (TIMP)-2 in the left posterior cerebral artery. The levels were determined by quantitative real time-PCR and normalized by the glyceraldehyde 3-phophate dehydrogenase (GAPDH) mRNA level. **c** Ratio of the MMP-9/TIMP-2 mRNA level. *p < 0.05 (Scheffe’s test). Data represent the mean ± SD. *n* = 7 per group

The mRNA level of MMP-9 was significantly higher in HT+OVX than HT rats. In contrast, the mRNA level of TIMP-2 was lower in HT+OVX than HT rats. The mRNA level of MMP-9 in BZA1 rats was significantly lower, and TIMP-2 was higher than in HT+OVX rats (Fig. 4a, b). Consequently, the MMP-9/TIMP-2 ratio was reduced in BZA1 rats (Fig. 4c). These observations suggest that elevated MMP-9 mRNA- and attenuated TIMP-2 mRNA expression are associated with the downregulation of ERs. The downregulation of IL-1β and the amelioration of the MMP/TIMP2 ratio via the modulation of ERs by BZA may have contributed to the prevention of aneurysmal rupture in our estrogen-deficient hypertensive rats.

## Discussion

We demonstrate that ovariectomy promoted aneurysmal rupture in hypertensive rats subjected to hemodynamic stress. We first document that in our hypertensive, ovariectomized aneurysm-model rats, the downregulation of ERs and the upregulation of pro-inflammatory molecules in the rupture-prone cerebral artery were associated with an increase in the aneurysmal rupture rate and that BZA ameliorated the downregulation of ERs, restored their gene and protein expression, decreased the incidence of aneurysmal rupture, and lowered the level of pro-inflammatory- and vascular degradation molecules. The incidence of ruptured aneurysms and the mRNA level of ERs and pro-inflammatory and vascular degradation molecules were similar to those in HT rats with intact ovaries. These findings suggest that BZA prevented aneurysmal rupture associated with inflammation and vascular degradation via the downregulation of ERs in our ovariectomized aneurysm-model rats.

Estrogen exerts diverse vascular effects mediated mainly by ERα and ERβ [23]. The treatment of ovariectomized rats with estradiol reversed the decrease in ERα and ERβ expression in the cerebral vessel wall [19]. Using our original aneurysm model, we demonstrated that estrogen deficiency elicited by ovariectomy reduced the expression of ERα while the expression of ERβ was retained at the bifurcation of the ACA-OA where unruptured aneurysms were highly and reproducibly induced [6]. The present study showed that the expression of ERα and ERβ in the PCA prone to aneurysmal rupture was reduced in our ovariectomized aneurysm rats and that BZA upregulated both ERα and ERβ in the PCA. Elsewhere, our group documented that in ovariectomized aneurysm mice, treatment with estrogen or an ERβ-, but not an ERα agonist, reduced the incidence of cerebral aneurysm rupture [9]. The downregulation of ERβ is thought to be associated with inflammation [24]. We suggest now that in our current rat aneurysm model, BZA exerted protective effects against rupture through ERβ stimulation.

IL-1β promotes extracellular matrix degradation by increasing the production of MMPs and decreasing the production of TIMPs [25, 26]. TNF-α and IL-6 also stimulate the expression of MMP-9 [27, 28]. In our current and earlier studies [18, 22], the increase in IL-1β and TNF-α and the imbalance between MMP-9 and TIMP-2 were associated with aneurysm rupture in ovariectomized aneurysm rats. BZA abated the increase in IL-1β and the imbalance between MMP-9 and TIMP-2 and resulted in a moderate inhibition of TNF-α. ERβ stimulation decreased cellular inflammasome activity and IL-1β expression after global cerebral ischemia in ovariectomized rats [24]. Selective ERβ agonists repress the transcription of pro-inflammatory genes including TNF-α [29]. The ERβ agonistic effects of BZA may be responsible for the downregulation of pro-inflammatory cytokines, resulting in the inhibition of vascular degradation.

Others reported that TNF-α modulates cerebral aneurysm formation and rupture. The TNF-α inhibitor 3,6’dithiothalidomide resulted in aneurysmal stabilization and a significant decrease in the rupture rate [30]. In contrast, a meta-analysis reiterated that IL-6 was not associated with the pathogenesis of intracranial aneurysms [31]. These studies support our present findings.

In women, ERα activation in the brain stimulated physical activity and reduced their food intake and body weight gain [32, 33]. Therefore, the effect of BZA at 1 mg/kg, but not 0.3 mg/kg, on the body weight of our rats may have involved the upregulation of ERα. On the other hand, since ERβ is thought to be associated with vasoprotection, the effect of BZA on the body weight via the upregulation of ERα may be smaller than the vasoprotective effects elicited by ERβ. Estradiol and a selective ERα agonist increased the uterine weight in mice [15]. However, BZA had no such effects in the current and an earlier study [20], indicating that its effects are tissue-specific.

Use of our original rat aneurysm model resulted in a high incidence (more than 80%) of unruptured aneurysms at the ACA-OA bifurcation; at most, 10% of these aneurysms ruptured during the 12-week observation period. For the current study, we modified the hemodynamics of our original model and produced a novel model in which approximately 50% of the experimentally induced aneurysms spontaneously ruptured [18]. With this new model, we demonstrated the protective effect of BZA, a selective ER modulator, on cerebral aneurysm rupture. The efficacy for prevention of aneurysm rupture may provide a new therapeutic option in the clinical setting.

Our study has some limitations. First, we assessed the changes in the mRNA level and the protein expression of the ERs elicited by ovariectomy and BZA. For a better understanding of the effects of individual ERs, studies using specific ER agonists or inhibitors and knockout animal are needed. Second, we started the administration of BZA 2 weeks after aneurysm induction without confirming the presence of cerebral aneurysms, and we did not confirm the presence of unruptured aneurysm in rupture-prone arteries at 12 weeks. Therefore, it is unclear whether BZA prevented their formation or their rupture. Third, the rodent age may affect their vascular response to estrogen [34], and menopause simulated by ovariectomy in our rats may be different from natural menopause in aged rats. Further studies are underway to address these questions.

## Conclusions

This is the first study to show that BZA exerted protective effects against aneurysmal rupture in hypertensive ovariectomized aneurysm rats without affecting their blood pressure. It ameliorated the downregulation of ERs, the upregulation of IL-1β, and the MMP-9/TIMP-2 imbalance in the cerebral artery. Since BZA has fewer estrogenic side effects than estrogen and is clinically used to address osteoporosis, it may help to prevent SAH in postmenopausal women.

## Additional files

Additional file 1: Figure S1. A ruptured aneurysm in the left posterior cerebral artery (PCA) of an OVX/VC rat (arrowhead). (TIFF 4425 kb)

Additional file 2: Figure S2. Wet uterine weight 5 weeks after aneurysm induction. *p < 0.05 (Scheffe’s test). Data represent the mean ± SD. *n* = 7 per group. (TIFF 2286 kb)

## Abbreviations

ACA-OA: Anterior cerebral artery-ophthalmic artery
BZA: Bazedoxifene
ER: Estrogen receptor
GAPDH: Glyceraldehyde 3-phosphate dehydrogenase
HT: Hypertension
IL: Interleukin
MMP: Matrix metalloproteinase
OVX: Ovariectomy
PCA: Posterior cerebral artery
qRT-PCR: Quantitative real-time PCR
SAH: Subarachnoid hemorrhage
SERM: Selective estrogen receptor modulator
TIMP-2: Tissue inhibitor of metalloproteinase-2
TNF: Tumor necrosis factor
