# Supplementary figures and images for: Bazedoxifene, a selective estrogen receptor modulator, reduces cerebral aneurysm rupture in Ovariectomized rats
Source: J Neuroinflammation. 2017 Oct 2;14:197. doi: 10.1186/s12974-017-0966-7 (PMC5625708; doi:10.1186/s12974-017-0966-7)

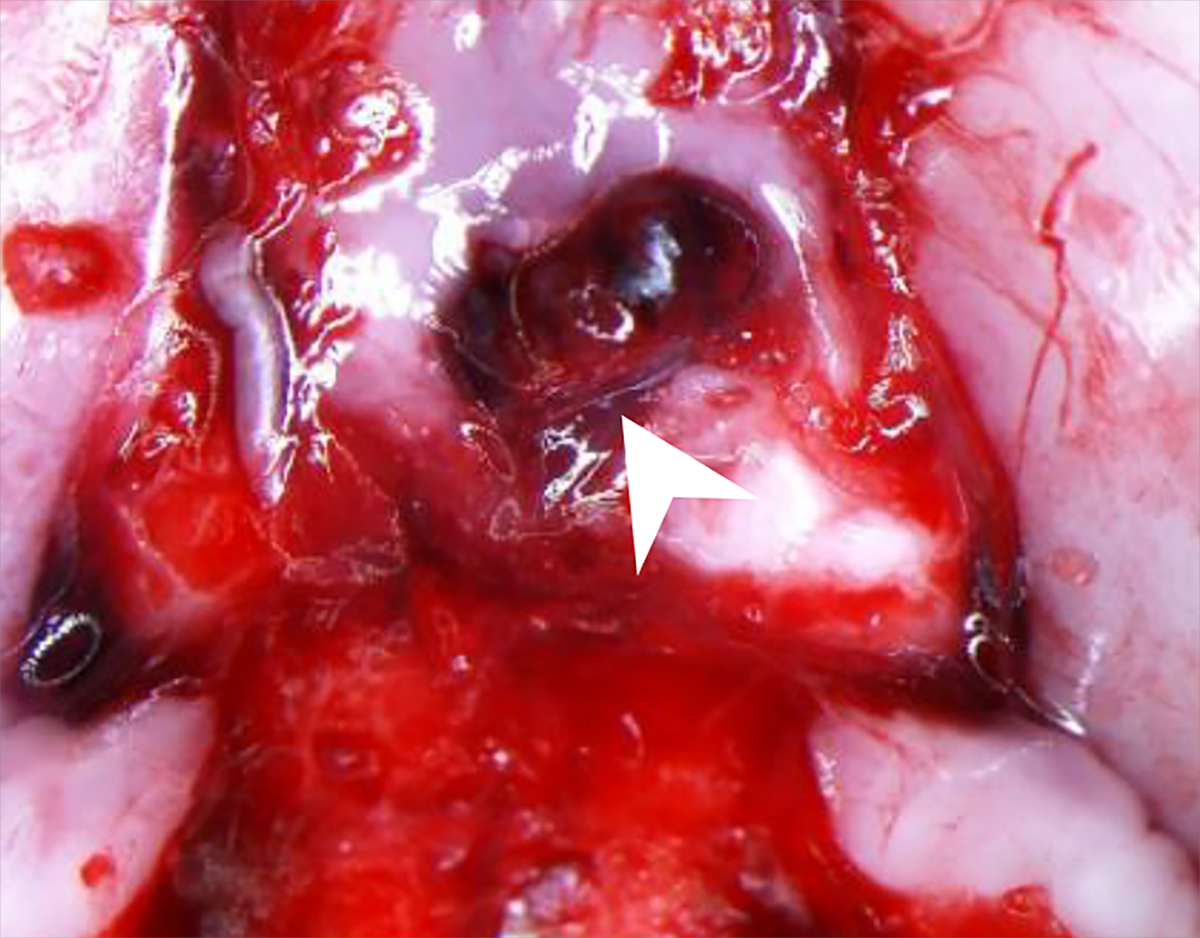

Supplement: Supplementary file 1 — A ruptured aneurysm in the left posterior cerebral artery (PCA) of an OVX/VC rat (arrowhead). (TIFF 4425 kb) [file 12974_2017_966_MOESM1_ESM.tiff]
